# Supplementary material for: Diagnostic status influences rapport and communicative behaviours in dyadic interactions between autistic and non-autistic people
Source: PLoS One. 2025 Aug 29;20(8):e0330222. doi: 10.1371/journal.pone.0330222 (PMC12396695; doi:10.1371/journal.pone.0330222)
Supplement: S1 File — (DOCX) [file pone.0330222.s001.docx]

## S1. Detailed Hypotheses and Mediation Models

Our hypotheses formed three clusters. The first cluster tested the effect of actor and partner diagnosis on rapport. This includes:

H1a: Autistic participants will report lower overall rapport compared to non-autistic participants.

H1b: Participants partnered with an autistic person will report lower rapport compared participants partnered with a non-autistic person.

H1c: Actor rapport will be lower when their partner has a different diagnostic status to them, compared to when they share the same diagnostic status.

The second cluster tests the relationship between participants diagnostic status and rapport, and examines whether this is mediated by their multimodal indices. This includes:

H2a: The relationship between an individual’s diagnostic status and rapport will be mediated by their multimodal indicess (Verbal Backchannel Rate, Nonverbal Backchannel Rate, Percent Laughing, Percent Smiling, and Mean Utterance Length, velocity, acceleration, and jerkiness). Specifically, autistic participants will exhibit lower levels of multimodal indicess and increased levels of kinematic indices (faster and jerkier movements), which in turn will be related to lower perceived rapport (e.g., see Figure 1 for this actor-actor pathway)

**Figure 1**

Mediation Model of Hypothesized Indirect Effects in Actor-Partner Pathways


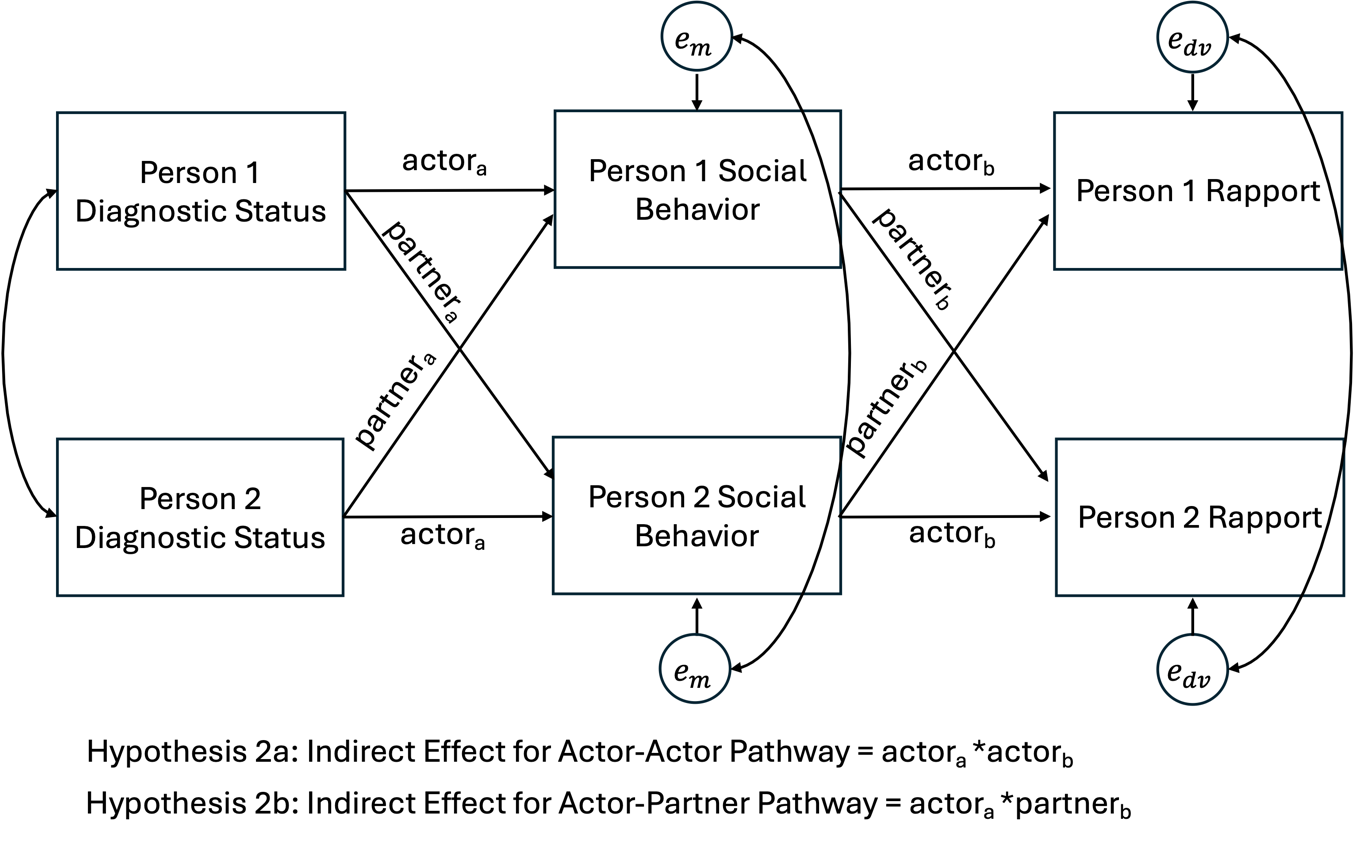


*Note*. The figure illustrates the hypothesised indirect effects for two pathways: the Actor-Actor Pathway (Hypothesis 2a) and the Actor-Partner Pathway (Hypothesis 2b). In the Actor-Actor Pathway, the indirect effect is the product of person 1 and person 2. In the Actor-Partner Pathway, the indirect effect is the product of person 1 and person 2. Diagnostic status, multimodal indices, and rapport for both Person 1 and Person 2 are considered within these pathways.

H2b: The relationship between a partner’s diagnostic status and the actors' rapport will be mediated by the partner’s multimodal indices. Specifically, when the partner is autistic, lower levels of multimodal indices and increased kinematic indices (faster and jerkier movements) will result in lower perceived rapport for the individual (see Figure 1 for the actor-partner pathway).

H2c: The mediation of the relationship between an individual’s diagnostic status and rapport by multimodal indices will differ depending on whether the actor and partner share the same diagnostic status. Specifically, we hypothesise that the mediation effect will be strongest in non-autistic dyads, as multimodal indices are more salient and relevant to their perceptions of rapport. In contrast, this mediation effect will be weaker in autistic dyads, where these indices may be less salient. Additionally, in mixed neurotype dyads, the mediation effect might fall between the two, with differing diagnostic statuses potentially heightening the influence of social and kinematic factors on rapport perceptions.

The third cluster examines the impact of an actor's diagnostic status on their multimodal indices (Verbal Backchannel Rate, Nonverbal Backchannel Rate, Percent Laughing, Percent Smiling, and Mean Utterance Length) and whether these moderated by both their diagnostic status and their partner's diagnostic status. This includes:

H3a: Individuals with an autistic diagnosis status will have lower indices of multimodal indices than compared to non-autistic individuals. Conversely, they will produce higher kinematic indices (velocity, jerk, acceleration).

H3b: Individuals whose partners have an autistic diagnostic status will have lower overall multimodal indices compared to those with non-autistic partners.

H3c: Actor multimodal indices will differ when their partner differs diagnostically compared to when they share the same diagnosis. Specifically, autistic actors are predicted to demonstrate more multimodal indices when paired with a non-autistic partner due to heightened social demands compared to when interacting with an autistic partner.
