# Supplementary material for: Diagnostic status influences rapport and communicative behaviours in dyadic interactions between autistic and non-autistic people
Source: PLoS One. 2025 Aug 29;20(8):e0330222. doi: 10.1371/journal.pone.0330222 (PMC12396695; doi:10.1371/journal.pone.0330222)
Supplement: S2 File — Materials presented to participants to measure rapport. (DOCX) [file pone.0330222.s002.docx]

## S2. Materials Presented to Participants to Measure Rapport

Rapport was measured using five items, with ratings measured using a visual analogue scale ranging from 0 to 100. The instructions were:


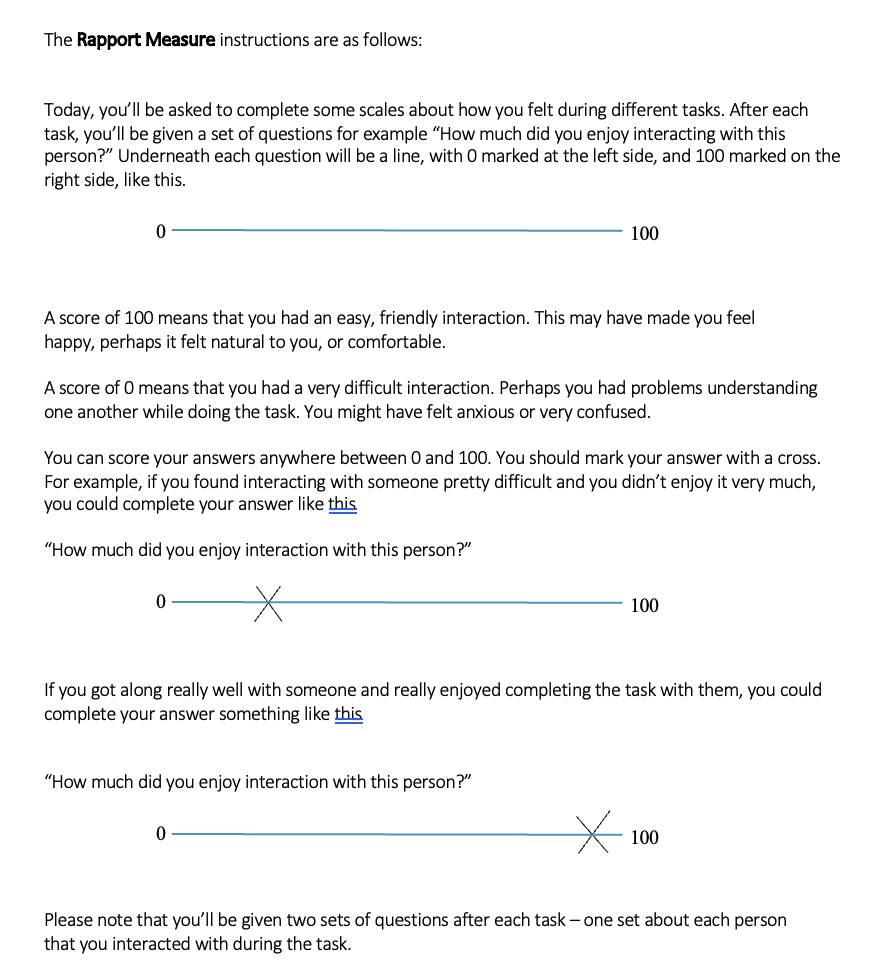


The five items were:

1. Enjoy: How much did you enjoy interacting with this person?
2. Easy: How easy did the current participant find interacting with the participant?
3. Successful: How successful did the current participant feel the interaction went with the participant?
4. Friendly: How friendly was the participant telling the current participant?
5. Awkward: How awkward was the interaction with the participant?
