# Supplementary material for: Diagnostic status influences rapport and communicative behaviours in dyadic interactions between autistic and non-autistic people
Source: PLoS One. 2025 Aug 29;20(8):e0330222. doi: 10.1371/journal.pone.0330222 (PMC12396695; doi:10.1371/journal.pone.0330222)
Supplement: S3 File — Dyad-level analysis on verbal/non-verbal and kinematic indices. (DOCX) [file pone.0330222.s003.docx]

**S3. Dyad level Analysis on** **verbal/non-verbal and kinematic indices**

We pre-registered an exploratory analysis to investigate whether dyad neurotype (autistic-autistic, non-autistic-non-autistic, or mixed neurotype pairs) influenced levels of verbal indices (Mean Utterance Length, Verbal Backchannel Rate), non-verbal indices (Nonverbal Backchannel Rate, Percent Laughing, Percent Smiling), and kinematic indices (velocity, acceleration, jerk). For each variable, we conducted a linear mixed-effects model with contrasts set to sum-to-zero coding (contr.sum) and reported the results using Type III ANOVA outputs.

**Table S1**

Results for the Effects of Neurotype and Blinding on Verbal, Nonverbal, and Kinematic Indices

| Variable | Effect | *df* | *F* | *p* |
| --- | --- | --- | --- | --- |
| Mean Utterance Length | Neurotype | 2, 43.05 | 2.07 | .14 |
| Mean Utterance Length | Blinding | 1, 47.09 | 0.01 | .94 |
| Mean Utterance Length | Interaction | 2, 46.83 | 1.10 | .34 |
| Verbal Backchannel Rate | Neurotype | 2, 42.34 | 1.60 | .21 |
| Verbal Backchannel Rate | Blinding | 1, 46.49 | 1.28 | .26 |
| Verbal Backchannel Rate | Interaction | 2, 46.22 | 2.05 | .14 |
| Nonverbal Backchannel Rate | Neurotype | 2, 42.86 | 0.75 | .48 |
| Nonverbal Backchannel Rate | Blinding | 1, 47.58 | 0.15 | .70 |
| Nonverbal Backchannel Rate | Interaction | 2, 47.28 | 3.24 | .048 |
| Percent Laughing | Neurotype | 2, 41.24 | 2.59 | .09 |
| Percent Laughing | Blinding | 1, 47.63 | 0.27 | .61 |
| Percent Laughing | Interaction | 2, 47.22 | 1.51 | .23 |
| Percent Smiling | Neurotype | 2, 42.85 | 0.95 | .40 |
| Percent Smiling | Blinding | 1, 51.63 | 0.34 | .56 |
| Percent Smiling | Interaction | 2, 51.24 | 0.00 | 1.00 |
| Velocity | Neurotype | 2, 43.27 | 1.72 | .19 |
| Velocity | Blinding | 1, 49.6 | 0.46 | .50 |
| Velocity | Interaction | 2, 49.21 | 0.67 | .52 |
| Acceleration | Neurotype | 2, 42.64 | 1.44 | .25 |
| Acceleration | Blinding | 1, 49.76 | 0.56 | .46 |
| Acceleration | Interaction | 2, 49.33 | 1.03 | .36 |
| Jerk | Neurotype | 2, 42.49 | 0.93 | .40 |
| Jerk | Blinding | 1, 49.76 | 0.88 | .35 |
| Jerk | Interaction | 2, 49.34 | 0.77 | .47 |

*Note.* This table presents the results of Type III ANOVA analyses examining the effects of neurotype, blinding, and their interaction on various verbal (e.g., mean utterance length, backchannel rates), nonverbal (e.g., percent laughing, percent smiling), and kinematic indices (velocity, acceleration, jerk).

The interaction breakdown for nonverbal backchannel rate reveals distinct patterns across neurotype groups. For autistic dyads, nonverbal backchannel rates were higher in the informed condition (*M* = 0.18, *SE* = 0.02, 95% CI [0.14, 0.22]) compared to the uninformed condition (*M* = 0.11, *SE* = 0.02, 95% CI [0.06, 0.16]), with a significant difference between conditions (estimate = 0.07, *SE* = 0.03, t(42) = 2.31, *p* = 0.026). In mixed dyads, nonverbal backchannel rates were slightly higher in the uninformed condition (*M* = 0.18, *SE* = 0.03, 95% CI [0.13, 0.23]) compared to the informed condition (*M* = 0.16, *SE* = 0.02, 95% CI [0.124, 0.20]), but this difference was not significant (*M* = -0.02, *SE* = 0.03, *t*(42) = -0.65, *p* = 0.521). For non-autistic dyads, nonverbal backchannel rates were slightly higher in the uninformed condition (M = 0.17, *SE* = 0.03, 95% CI [0.11, 0.22]) compared to the informed condition (*M* = 0.14, *SE* = 0.02, 95% CI [0.10, 0.17]), though this difference was also not significant (*M* = -0.03, *SE* = 0.03, *t*(61.7) = -0.93, *p* = 0.355). These results suggest that the informed condition specifically enhances nonverbal backchannel rates for autistic dyads, while no significant effects were observed in mixed or non-autistic dyads.
