# Supplementary material for: Diagnostic status influences rapport and communicative behaviours in dyadic interactions between autistic and non-autistic people
Source: PLoS One. 2025 Aug 29;20(8):e0330222. doi: 10.1371/journal.pone.0330222 (PMC12396695; doi:10.1371/journal.pone.0330222)
Supplement: S4 File — Analysis of kinematic indices by joint. (DOCX) [file pone.0330222.s004.docx]

**S4. Analysis of kinematic indices by joint**

**Table S2**

ANOVA Results for the Effects of Joint and Neurotype on Acceleration, Velocity, and Jerk

| Variable | Effect | *df* | *F* | *p* |
| --- | --- | --- | --- | --- |
| Acceleration | Joint | 3, 349.77 | 11.58 | < .001 |
| Acceleration | Neurotype | 2, 43.43 | 2.00 | .15 |
| Acceleration | Joint:Neurotype | 6, 349.77 | 1.19 | .31 |
| Velocity | Joint | 3, 349.91 | 11.56 | < .001 |
| Velocity | Neurotype | 2, 43.61 | 2.11 | .13 |
| Velocity | Joint:Neurotype | 6, 349.91 | 1.25 | .28 |
| Jerk | Joint | 3, Inf | 12.35 | < .001 |
| Jerk | Neurotype | 2, 44 | 1.90 | .16 |
| Jerk | Joint:Neurotype | 6, Inf | 1.14 | .34 |

*Note.* This table presents the results of Type III ANOVA analyses examining the effects of joint, neurotype, and their interaction on acceleration, velocity, and jerk. These analyses used Satterthwaite’s method for estimating degrees of freedom.

The main effect of joint Post hoc analyses for the main effect of joint reveal consistent patterns across metrics (acceleration, velocity, and jerk). For all three measures, the right arm (rarm) exhibited significantly higher values compared to the left arm (arm), head, and torso. The left arm also showed higher values than the head and torso, with significant differences for comparisons involving the torso. The head and torso did not significantly differ across any measure. These results highlight that the arms, particularly the right arm, exhibit the highest values across metrics, while the head and torso show lower and relatively comparable values. However, it is important to note that these effects may be influenced by interactions with neurotypes, which could complicate interpretation.
