# Supplementary material for: Diagnostic status influences rapport and communicative behaviours in dyadic interactions between autistic and non-autistic people
Source: PLoS One. 2025 Aug 29;20(8):e0330222. doi: 10.1371/journal.pone.0330222 (PMC12396695; doi:10.1371/journal.pone.0330222)
Supplement: S5 File — Details on analogous Bayesian models. (DOCX) [file pone.0330222.s005.docx]

**S5. Analogous Bayesian Models**

For Bayesian multilevel modelling, we utilised the *brms* package[1] and for SEMs we used *blaavan*[2]. We used flat priors in our model to allow the data to drive the parameter estimates without imposing any prior assumptions. This is appropriate given the lack of strong prior information and the goal of minimising subjective influence on the results. For the analysis on rapport scores, we used a skew-normal distribution for the analysis on rapport.

*H1*

The equivalent Bayesian mixed model supported the frequentist, indicating decreased rapport when the actors’ diagnostic status was autistic compared to non-autistic (*b* = −25.68, *SE* = 8.76, 95% Credible Interval [−43.23 −8.39]). Further, it corroborated H1b that is no partner effect, as the credible interval containing zero (*b* = 8.01, *SE* = 8.50, 95% Credible Interval [−8.86, 24.93]). Finally, it did not support H1c, indicating a lack of interaction between partner and actor diagnostic status (*b* = −3.01, *SE* = 8.93, 95% Credible Interval [−20.57 14.77]).

*H2*

The Bayesian mediation analyses, mostly, support the frequentist (see Table 1). However, the Bayesian the Bayesian results suggest a potential actor-actor pathway (H2a) where autistic individuals may experience reduced rapport due to fewer verbal backchannels (*b* = -28.79; Credible Interval [-50.41 -7.17). Additionally, a positive actor-actor effect for mean utterance length suggests that longer speaking turns may enhance autistic participants own self-reported rapport (*b* = 29.44; Credible Interval [15.00, 43.87]). Finally, an actor-partner pathway (H2b) suggests that non-autistic partners’ backchanneling behaviour can positively impact autistic actors’ sense of rapport (*b* = 22.38; Credible Interval [0.90, 43.80]). These findings, though exploratory and requiring cautious interpretation, indicate the presence of an effect that may have been too subtle for detection in the frequentist analysis.

**Table S3**

Indirect Effects of Multimodal Indices on the Relationship Between Diagnostic Status and Rapport

| Hypothesis | *b* | 95% Credible Interval |
| --- | --- | --- |
| H2a |  |  |
| Verbal Backchannels | -28.79 | [-50.41 -7.17] |
| Nonverbal Backchannels | -14.57 | [-36.02 6.89] |
| Percent Laughing | -2.47 | [-20.54 15.61] |
| Percent Smiling | 7.58 | [-3.74 18.89] |
| Mean Utterance Length | 29.44 | [15.00 43.87] |
| Acceleration | 0.38 | [-2.02 2.78] |
| Velocity |  |  |
| Jerkiness | -0.11 | [-2.07 1.85] |
| H2b |  |  |
| Verbal Backchannels | 22.64 | [1.33 43.96] |
| Nonverbal Backchannels | 20.40 | [-1.06 41.86] |
| Percent Laughing | -8.76 | [-26.95 9.43] |
| Percent Smiling | 3.07 | [-8.14 14.27] |
| Mean Utterance Length | -6.61 | [-20.31 7.10] |
| Acceleration | 0.25 | [-2.25, 2.75] |
| Velocity |  |  |
| Jerkiness | -0.12 | [-2.12, 1.89] |
| H2c (Actor-Actor Pathway) |  |  |
| Verbal Backchannels | -4.21 | [-39.58 31.15] |
| Nonverbal Backchannels | -15.06 | [-51.12 21.01] |
| Percent Laughing | -32.57 | [-77.38 12.23] |
| Percent Smiling | 26.15 | [-5.05 57.34] |
| Mean Utterance Length | -1.09 | [-30.12 27.94] |
| Acceleration | -1.36, | [-6.11 3.39] |
| Velocity |  |  |
| Jerkiness | 1.10 | [-2.94 5.132] |
| H2c (Actor-Partner Pathway) |  |  |
| Verbal Backchannels | -3.82 | [-35.86 28.22] |
| Nonverbal Backchannels | -13.59 | [-46.22 19.05] |
| Percent Laughing | -34.63 | [-82.11 12.85] |
| Percent Smiling | 18.59 | [-4.26 41.44] |
| Mean Utterance Length | -1.26 | [-34.96 32.43] |
| Acceleration | -1.29 | [-6.20 3.61] |
| Velocity |  |  |
| Jerkiness | 1.13 | [-3.01, 5.26] |

*Note*. The table shows the indirect effects of various multimodal indices on the relationship between diagnostic status and rapport. The Bayesian SEM for the outcome Velocity did not converge.

*H3*

The Bayesian mixed models reproduced the frequentist mixed models (see table 2), however the size of the effects differed due to the family specification (e.g. log/skew family distribution).

The Bayesian analysis revealed that autistic participants exhibited, on average, a 0.15 lower backchannel rate (measured as the number of backchannels per minute of listening time) compared to the non-autistic group. Given that most participants’ rates ranged between 0.1 and 0.2 backchannels per minute, with a maximum of 0.46, this reduction appears substantial. However, the credible interval for the beta coefficient (Credible Interval [-0.29 to -0.01]) indicates some uncertainty.

Similarly, for mean utterance length, both approaches indicated that autistic participants exhibited significantly longer mean utterance lengths compared to non-autistic participants. The Bayesian analysis, using a lognormal distribution to account for the positive and right-skewed nature of the data (*b* = 0.19, Credible Interval [0.09, 0.29]) indicates an approximate 20.9% increase in mean utterance length for autistic participants compared with non-autistic participants.

**Table S4**

APIM Results for Hypothesis 3

| Outcome Variable | Predictor | *b* | *SE* | 95% CI |
| --- | --- | --- | --- | --- |
| Verbal  Backchannel Rate | Actor (H3a) | -0.15 | 0.08 | [-0.29  -0.01] |
|  | Partner (H3b) | -0.02 | 0.07 | [-0.16  0.12] |
|  | Interaction (H3c) | -0.05 | 0.07 | [-0.18 0.10] |
| Nonverbal  Backchannel Rate | Actor (H3a) | 0.05 | 0.07 | [-0.09 0.19] |
|  | Partner (H3b) | -0.03 | 0.07 | [-0.17 0.11] |
|  | Interaction (H3c) | 0.09 | 0.07 | [-0.23 0.07] |
| Percent Laughing | Actor(H3a) | -0.04 | 0.08 | -0.20] [0.12 |
|  | Partner (H3b) | -0.10 | 0.08 | -0.27] [0.07] |
|  | Interaction (H3c) | -0.11 | 0.10 | [-0.32 0.08] |
| Percent Smiling | Actor(H3a) | 0.12 | 0.11 | [-0.11 0.34] |
|  | Partner (H3b) | 0.07 | 0.12 | [-0.16 0.30] |
|  | Interaction (H3c) | 0.16 | 0.15 | [-0.14 0.46] |
| Mean Utterance Length | Actor (H3a) | 0.19 | 0.05 | [0.09 0.29] |
|  | Partner (H3b) | -0.03 | 0.05 | [-0.13 [0.08] |
|  | Interaction (H3c) | 0.01 | 0.06 | [-0.10 0.12] |
| Acceleration | Actor(H3a) | 0.19 | 2.04 | [-3.91 4.12] |
|  | Partner (H3b) | -0.56 | 2.10 | [-4.53 3.71] |
|  | Interaction (H3c) | -4.84 | 2.73 | [-10.51 0.38] |
| Velocity | Actor(H3a) | 0.38 | 0.58 | [-0.79 1.47] |
|  | Partner (H3b) | -0.70 | 0.59 | [-1.85 0.47] |
|  | Interaction (H3c) | -1.05 | 0.67 | [-2.46 0.17] |
| Jerkiness | Actor(H3a) | -0.10 | 66.34 | [-131.97 130.90] |
|  | Partner (H3b) | 13.59 | 66.10 | [-113.80 146.77] |
|  | Interaction (H3c) | -136.53 | 90.64 | [-319.84 36.64] |

*Note.* The table presents the estimated effects of actor diagnostic status on various behaviours. The beta coefficients represent the direction and magnitude of the difference between autistic (coded as 1) and non-autistic (coded as -1) participants in each model. Positive beta values indicate that autistic participants exhibit higher values for the respective behaviour compared to non-autistic participants, while negative values suggest lower values. All models include dyad ID as a random effect to account for shared variance within dyads. Grey shaded cells indicate credible intervals excluding zero.

**References**

1. Bürkner P-C. brms: An R Package for Bayesian Multilevel Models Using Stan. Journal of Statistical Software. 2017;80: 1–28. doi:10.18637/jss.v080.i01

2. Merkle EC, Rosseel Y. blavaan: Bayesian structural equation models via parameter expansion. arXiv; 2016. doi:10.48550/arXiv.1511.05604
