# Supplementary material for: Diagnostic status influences rapport and communicative behaviours in dyadic interactions between autistic and non-autistic people
Source: PLoS One. 2025 Aug 29;20(8):e0330222. doi: 10.1371/journal.pone.0330222 (PMC12396695; doi:10.1371/journal.pone.0330222)
Supplement: S6 File — (DOCX) [file pone.0330222.s006.docx]

**S6. Coding Scheme**

Table S6

Coding Scheme

| Variable | Inclusion Criteria | Exclusion Criteria |
| --- | --- | --- |
| Utterance Length (duration, seconds) | Duration of speech produced by a participant in each interactive turn, measured in seconds (to three decimal places). Includes pauses that do not indicate the end of the speaker’s turn (see Number of Turns Taken). | Pauses that do indicate the end of the speaker’s turn (see Number of Turns Taken). |
| Mean Utterance Length (seconds) | Sum of all utterance lengths for a participant divided by the number of turns taken. | – |
| Maximum Utterance Length (seconds) | The longest utterance produced by a participant. | – |
| Minimum Utterance Length (seconds) | The shortest utterance produced by a participant. | – |
| Verbal Backchanneling (frequency) | Verbal cues indicating attentiveness and/or comprehension without the intention to interrupt. Includes: non-lexical elements/minimal vocalisations (e.g., “mhm”, “uhhuh”); short lexical elements (e.g., “wow”); and brief comments (e.g., “Yeah”, “Right”, “Oh yeah”, “Oh I see”). | Any verbal signal that disrupts the speaker’s turn, such as: questions requesting a response (typically with a rising tone, including clarifications); comments intended to interrupt or redirect the conversation (including unsuccessful interruptions); and any backchannel produced by a non-listener. Additionally, exclude repeated words/phrases, finishing the speaker’s utterance, gasps (unless otherwise classifiable as backchannels), and co-occurring laughs (where the laugh itself is not counted as a backchannel). |
| Nonverbal Backchanneling (frequency) | Nonverbal cues such as nodding and shaking the head that indicate attentiveness. | Head tilts, facial expressions (e.g., smiles), hand gestures, or any nonverbal signal that does not convey attentiveness (e.g., if produced by a non-listener). |
| Verbal Backchanneling Rate (per minute of listening) | Calculated as the number of verbal backchannels divided by the time spent listening (total interaction time minus total speaking time). | – |
| Nonverbal Backchanneling Rate (per minute of listening) | Calculated as the number of nonverbal backchannels divided by the time spent listening (total interaction time minus total speaking time). | – |
| Smile Duration (seconds) | Duration (to three decimal places) of a single smile, defined as the period during which the lip corners pull up (often with raised cheeks and eye/eyebrow muscles). Includes subtle smiles and those involving one side of the face; may be with an open or closed mouth; may co-occur with speech or occur during silence. Ends at the transition to a non-smile expression. If a smile continues after a laugh, it is coded as a separate smile beginning after the laugh; if a smile precedes a laughing smile, its duration is measured up to the start of the laughing smile/laugh. | Non-smiling expressions (e.g., lip presses, frowns, surprised looks); laughing smiles (i.e., smiles co-occurring with laughter, which should be coded as laughter); and suspected smiles where the mouth is obscured for the entire duration. |
| Smile Frequency | Total number of smiles exhibited by a participant during the interaction. A smile is defined by the visible portion of the face; if the lower half is temporarily obscured (e.g., by a hand), end the current smile and begin a new one when visibility resumes. | – |
| Mean Smile Duration (seconds) | Total smile duration divided by the number of smiles. | – |
| Percent Smiling | The percentage of the interaction time during which a participant is smiling, calculated by dividing the total smile duration by the total interaction time. | – |
| Laugh Duration (seconds) | Duration (to three decimal places) of a laugh, identified via audible or physical indications. Includes: audible laughter (e.g., repeated “ha ha”) and laughter inferred from repeated air expulsions and/or torso movements. Laughs are separated by a break of more than 1 second; each laugh begins at the first indication and ends at an intake of breath or when all laughter indicators cease. If a laughing smile co-occurs, the laugh ends when the movement/sound ceases or the final intake of breath occurs. | Sarcastic laughter imitations (e.g., flat, ironic “ha – ha – ha”); torso movements not indicating laughter (e.g., coughing); and large, open-mouthed smiles that occur without accompanying audible or physical laughter. |
