# Supplementary material for: Diagnostic status influences rapport and communicative behaviours in dyadic interactions between autistic and non-autistic people
Source: PLoS One. 2025 Aug 29;20(8):e0330222. doi: 10.1371/journal.pone.0330222 (PMC12396695; doi:10.1371/journal.pone.0330222)
Supplement: S7 File — (DOCX) [file pone.0330222.s007.docx]

## Deviations from Pre-Registration

Analyses for this study were registered at (https://osf.io/tmuqn/). We pre-registered the use of the variables “non-verbal back-channel rate” and “verbal back-channel rate” for our mediation models, which were calculated by dividing the total number of verbal backchannels (frequency) by the total time the participant spent listening (interaction time minus speaking time). However, during the analysis, we encountered convergence issues due to significant discrepancies in the variances of our observed variables, specifically, some variables had variances that were at least 1,000 times larger than others. This large variance disparity led to numerical instability in the model estimation process. To address this issue and achieve reliable convergence, we opted to use the total counts of verbal and non-verbal back channels instead of their rates.
