# Supplementary material for: Diagnostic status influences rapport and communicative behaviours in dyadic interactions between autistic and non-autistic people
Source: PLoS One. 2025 Aug 29;20(8):e0330222. doi: 10.1371/journal.pone.0330222 (PMC12396695; doi:10.1371/journal.pone.0330222)
Supplement: Attachment — Submitted filename: review_PONE-D-25-10256.pdf [file pone.0330222.s008.pdf]

Thank you for the opportunity to review this article. This is a well-written paper presenting a thoughtfully designed study that makes a valuable contribution to the field. The comprehensive analysis of verbal and non-verbal cues across same- and mixed-neurotype interactions addresses an important gap in the existing literature. I offer the following suggestions to enhance the paper's clarity.

Page 3, line 57: I find this sentence unclear. If a shift is mentioned, I would expect some contrast between views/methodologies to be present in the previous sentences, which is not the case.

Page 6, lines 132-133: This sentence is slightly misleading. When first reading it, I assumed that utterance length had been measured in number of words, since it is introduced as a verbal interaction metric. However, the supplementary material reveals that length was measured in seconds, including silent pauses—which constitutes a non-verbal component. To avoid confusion, I suggest reformulating this sentence and/or specifying upfront that utterance length refers to the temporal duration of utterances.

I would also like to see a brief explanation of 1) why utterance length was chosen as an interaction metric (utterance length is not mentioned in the Introduction, whereas other measures such as smiles, nods, movements are) and 2) why the authors chose to measure it in seconds rather than in words ?

Page 6, line 136: I also wonder if referring to the eight elements of social interaction as multimodal indices is correct since most elements are not multimodal but unimodal (e.g., nods, smiling, total upper body velocity, acceleration, and jerkiness).

Page 7, lines 144-148: I find the formulation of hypothesis 1(and its sub-hypotheses) unclear and would suggest reformulating as separate sentences or as bullet points. Furthermore, it is not clear from the introduction and literature review why the authors hypothesise that self-reported rapport will be lower for autistic participants compared to non-autistic participants.

Page 7, lines 149-164: Hypothesis 2a mentions social behaviors, are those the same as multimodal indices mentioned in Hypothesis 3? If yes, why use two different terms? If not, what does social behaviors refer to?

Likewise, Hypothesis 2a stipulates that autistic individuals will produce increased, less smooth movements but Hypothesis 3a stipulates that they will exhibit fewer multimodal indices (which includes kinematic measures as stated on page 6 (lines 134-135) in the research aims). This seems contradictory.

Finally, the authors expect autistic participants to increase social behaviours such as smiling in mixed neurotype dyads due to heightened social demands (H3c). What does heightened social demands refer to specifically?

I would suggest reformulating more clearly and consistently hypothesis 2 and 3 (and their sub-hypotheses).

Page 8, lines 185-186: The inclusion of self-diagnosed individuals in the autistic group should be mentioned upfront in the abstract. While I fully support including self-identified participants—given recruitment challenges and barriers to formal diagnosis—this information needs to be transparent from the outset. Group composition impacts result interpretation and generalizability and should also be discussed in the Discussion/Limitation section.

Related to this, I also wonder if it is appropriate to use “diagnostic status” as outcome variable name, given the mixed status of participants. Maybe a preliminary note would be useful to clarify what the authors mean by diagnostic status.

I'd also recommend reporting the distribution of clinical versus self-diagnosis across dyad types, not just by individual group membership.

Page 8, line 189: Could the authors provide more information on how dyad partners were matched. Did they try to match them by age or sex/gender ?

Page 11, line 254: What were the causes of coding disagreement for the variable “Smiling” ? Were they systematic or random ? This result should also be mentioned in the Discussion or Limitation section.

Typos

Page 5, 99-99: To address this gap, the Actor-Partner Interdependence Model (APIM) ~~is a~~ is a statistical model used to analyse data from dyadic relationships which allows for the simultaneous examination of how each person's behaviours and characteristics influence not only their outcomes but those of their partner.

Page 12, line 276: recording → recoding
