# Supplementary material for: Diagnostic status influences rapport and communicative behaviours in dyadic interactions between autistic and non-autistic people
Source: PLoS One. 2025 Aug 29;20(8):e0330222. doi: 10.1371/journal.pone.0330222 (PMC12396695; doi:10.1371/journal.pone.0330222)
Supplement: Attachment — Submitted filename: Response_to_reviewer.docx [file pone.0330222.s009.docx]

**Journal Requirements**

**1. Formatting:** "Please ensure that your manuscript meets PLOS ONE's style requirements, including those for file naming."

**Response:** We have reviewed the manuscript and confirm that it now adheres to the PLOS ONE style and formatting guidelines. The files have been named according to the journal's requirements.

**2. Ethics (Consent):** "Please describe in your methods section how capacity to provide consent was determined for the participants in this study. Please also state whether your ethics committee or IRB approved this consent procedure. If you did not assess capacity to consent please briefly outline why this was not necessary in this case."

**Response:** We did not assess capacity to consent for this study, as this was not deemed necessary by our ethics committee and IRB. As the paper states, *“Participants were recruited via autism support groups, community centres, via social media, and through networks across three Universities.”* All participants were able to independently contact the researcher, fill in an online form, and attend a research day. Thus, it was assumed, that participants had capacity to choose to take part in this research.

**3. Financial Disclosure:** "Please state what role the funders took in the study. If the funders had no role, please state: “

**Response:** The requested statement has been added to our cover letter. The funders had no role in study design, data collection and analysis, decision to publish, or preparation of the manuscript. However, the foundation offered additional funding to allow us to include a condition in which participants were uninformed about the diagnostic status of their interaction partner.

**4. Ethics Statement Location:** "Your ethics statement should only appear in the Methods section of your manuscript. If your ethics statement is written in any section besides the Methods, please delete it from any other section."

**Response:** We have confirmed that the ethics statement now appears only in the Methods section and removed it from page 31, lines 702-707.

**5. Figure 1 Copyright:** "We note that Figure 1 in your submission contain copyrighted image... We require you to either (1) present written permission from the copyright holder... or (2) remove the figures from your submission."

**Response:** Thank you for this query. Figure 1 is an original image created by the authors for this manuscript. It is a single frame from the videos used as part of this study; participants explicitly provided consent for their image to be used for research purposes. We own the copyright and grant PLOS ONE permission to publish it under the Creative Commons Attribution (CC BY 4.0) license.

**6. Table 4 Reference:** "Please ensure that you refer to Table 4 in your text; if accepted, production will need this reference to link the reader to the Table."

**Response:** A reference to Table 4 has now been added to the main text page 18 line 352.

**7. Supporting Information:** "Please include captions for your Supporting Information files at the end of your manuscript, and update any in-text citations to match accordingly."

**Response:** We have added captions for all Supporting Information files at the end of the manuscript and ensured all in-text citations are correct.

**8. Data Anonymization:** "Please remove all personal information, ensure that the data shared are in accordance with participant consent, and re-upload a fully anonymized data set."

**Response:** The dataset has been fully anonymised, with all personal information removed, and has been re-uploaded, these can be found on the Open Science Framework (OSF): <https://osf.io/tmuqn/>.

**Editor's Comments**

**1. General Comment:** "Several aspects require further refinement and clarification. These include details regarding the research methodology, such as the formulation of hypotheses, the rationale behind the choice of indices, and a description of the process for matching dyad partners, as well as certain unclear sections in the introduction and discussion, as highlighted in the reviews."

**Response:** We have comprehensively revised the manuscript to address these points. Specifically, we have reformulated the hypotheses for clarity, provided a detailed rationale for our chosen indices, expanded the description of the dyad matching process, and clarified the noted sections in the Introduction and Discussion. Detailed responses to the specific reviewer comments on these points are provided below.

**Reviewer Comments**

Reviewer #1: The manuscript in question tackles an intriguing question using a novel and appropriate approach. It was also enjoyable to read. The authors are correct that this area is currently understudied and I believe their report will present a valuable addition to the study of Autism and Social Cognition. Nonetheless, I have a few thoughts I would like to offer for consideration before I can recommend this article for publication.

**Response:** We thank the reviewer for their time and feedback on our manuscript.

The introduction is very informative and focused, but I'd like to see a brief explanation/definition of the terms rapport and backchanneling before these are used in the argument - the former is sometimes ambiguous and can be operationalised in different ways, whereas the latter may not be known to many readers. The role of kinematics and why they matter when exploring rapport and comunication could also be established more as that is another non-intuitive factor.

**Response:** We agree that defining these key terms improves the manuscript’s clarity. Accordingly, we have revised the definition of ‘rapport’ in the introduction for greater precision. On page 3, lines 43-46, the text now states: “Social interactions are important for developing rapport, defined as a state of harmonious connection and mutual understanding between individuals[1,2]. This connection is developed through the intricate interplay of verbal and nonverbal communication and is crucial for forming social bonds and enhancing well-being[3–9].”

We have also added a definition for 'backchanneling' on page 4, lines 101-102, the text now includes the following: “...examining markers of rapport (mutual gaze and backchanneling, i.e., verbal and non-verbal cues like nodding or saying ‘uh-huh’ to show engagement)...”

I would also appreciate if the authors could briefly cover the deficits-or-differences argument, as this is very relevant here. The authors already bring up the current implicit understanding of Autism in terms of social deficits (line 59) that contrasts with the cited literature showing effective communication and high rapport among matched pairs. I would just like to see this addressed explciitly.

**Response:** Thank you for this suggestion. We have added the following to page 3, lines 61-64, which now reads: ‘Over recent years, autistic social traits have been increasingly interpreted as being “different” rather than “deficient”; that is, that autistic social communication is a natural variation in interactive styles rather than something that needs to be corrected or mitigated [29]. ‘

The authors mention larger group setting (line 92) and groups larger than dyads (line 114) - briefly addressing dyadic vs group interactions may be informative and clarify the scope of the current manuscript.

**Response:** We thank the reviewer for this important point about clarifying the manuscript's scope. We agree that the mention of larger groups could be confusing. To address this, we have made two changes: 1. On page 5 lines 107-115, we have removed the phrase “particularly in larger group setting” to keep the focus clearly on dyadic interactions. It now reads: “This finding further highlights the unique dynamics present in same-neurotype interactions, compared to mixed-neurotype interactions[10]. Together, these studies emphasise the need for deeper investigation into the interactional nuances within mixed and matched neurotype pairs.”

Where the use of APIM in various contexts is mentioned (line 114), we have added a sentence to reinforce the scope of the current study and justify our focus on dyads on page 6 lines 146-149, which now reads: “As dyadic interactions are the foundational building blocks of group dynamics, the present study focuses on two-person pairings to isolate these core interpersonal behaviours before they are compounded by the complexities of a group setting.”

For understandable reasons, the introduction and article in general treat "Autistic" and "Non-Autistic" as homogenous groups. It is okay and expected if intra-group diversity is not within scope, but this is a broad issue within the field at large so it should be explicitly stated as a limitation: Both co-occuring conditions, and different levels of Autism (certainly level 3) - would no-doubt affect the results and dyadic communication, as would other individual differences and conditions among the non-Autism group that were not recorded.

**Response:** We absolutely agree that both “autistic” and “non-autistic” are heterogenous, diverse groups. Autistic diagnostic status is just one of many factors that may affect how someone interacts and behaves. However, these were the comparison groups of interest in this study, and so this was our focus in the write up. We agree that intellectual disability, in either autistic or non-autistic participants, may affect results, and have now included in the limitations section on page 23, lines 607-608: “Fourth, our participants had relative high IQs, and as such may not be representative of the wider population.”

I notice that participants were recruited from different countries. Could the authors please clarify whether each country contributed the sae proportion to each dyad-arrangement? It doesn't have to be equal between countries but it should be equal across arrangements. If any country contributed, for example, a majority of Autistic dyads but a minority of Non-Autistic dyads, then cultural factors become an important consideration.

**Response:** Thank you for this important question. To ensure the recruitment site did not act as a confounding variable, we performed a Pearson's Chi-squared test of independence. The analysis showed no significant association between recruitment site and dyad type distribution (χ²(4) = 1.03, p = .91), indicating the proportions were not significantly different across sites. We have added a note to the manuscript to clarify this point. On page 8-9, lines 234-236, the text now states: ". To confirm that the distribution of dyad types was comparable across the three Universities, a Pearson's Chi-squared test of independence was performed. The analysis showed no significant association between recruitment site and dyad type (χ²(4) = 1.03, p = .91), indicating the groups were sufficiently balanced in this regard”. The raw counts are as follows:

Dallas: Autistic (n=12), Mixed (n=10), Non-autistic (n=12)
Edinburgh: Autistic (n=16), Mixed (n=12), Non-autistic (n=10)
Nottingham: Autistic (n=12), Mixed (n=12), Non-autistic (n=12)

Might the results regarding the diagnostic status of the partner be influenced by social desirability? It seems logical that one dyad member would be hesitant to report low rapport if they knew their partner was Autistic, which would explain the high rapport of mixed dyads. This would be an interesting effect in itself but would present a confound that should be mentioned. To further explore this, the authors could compare the rapport ratings within mixed pairs, looking for differencxes between the Autistic and non-Autistic partner's ratings.

I may be reading this wrong (which may affect my previous point), but in the paragraph starting on line 362, the authors seem to say that informed mixed dyads report higher rapport, which is driven by the Autistic member who, when informed, reports lower rapport. This is obviously contradictory so I am guessing something may have gotten switched around here or the paragraph is not entirely clear. Upon reviewing figure 3 and table 4, I think there is a mistake in line 363, which possibly should say that uninformed mixed dyads reported higher rapport, not lower. In that case my previous point becomes moot but I would still like to see a deeper discussion of this effect.

**Response:** Here we respond to the two previous comments. Thank you for your careful review and for identifying this error. You are absolutely correct - there was a mistake in our original text. The corrected statement should read that uninformed mixed dyads reported higher rapport than informed mixed dyads, not the reverse as we mistakenly wrote. We have made this correction on pages 18-19, lines 406-506.

The accurate interpretation is that when mixed dyads are uninformed of diagnostic status, they report significantly higher rapport (*M_diff_* = -81.1, *p* = .015, Cohen's *d* = -1.16), and as you correctly noted from our descriptive statistics, this effect appears to be driven by the autistic member who reports substantially higher rapport when uninformed (*M* = 447) compared to informed (*M* = 365.82) of their partner's neurotypical status.

This correction actually strengthens rather than undermines the interpretation, as it aligns with our central finding that autistic individuals are more sensitive to diagnostic awareness than to neurotype composition per se. The pattern now shows that autistic individuals benefit from non-disclosure when interacting with neurotypical partners, but benefit from disclosure when interacting with other autistic individuals - suggesting different optimal strategies depending on interaction context rather than a simple social desirability bias.

Regarding your social desirability concern, while this remains a theoretical possibility, the corrected pattern actually argues against this interpretation. If social desirability were the primary driver, we would expect consistently higher rapport ratings when diagnostic status is known (to avoid appearing prejudiced). Instead, we observe a more complex interaction where diagnostic awareness helps in some contexts (autistic dyads) but hinders in others (mixed dyads), recommending genuine differences in social experience rather than response bias.

We appreciate your thorough review, which helped us identify and correct this important error while clarifying the theoretical implications of our findings.

It is already part of the data but a direct test comparing ratings of Autistic participants, depending on whether they interacted with an Autistic or non-Autistic partner, may be interesting. Currently this is somewhat hard to judge due to mixed pairs being averaged across the two participants.

**Response:** We thank the reviewer for this suggestion. We conducted a direct comparison of rapport ratings from autistic participants in same-neurotype dyads versus those in mixed-neurotype dyads. An independent-samples t-test revealed no significant difference in self-reported rapport between these two groups, *t*(33.02) = 0.38, *p* = .71. This suggests that for autistic participants in our sample, their perception of rapport was not significantly influenced by the neurotype of their interaction partner. We have added this analysis to the manuscript.

Minor comments:

On line 96, the words "is a" are repeated.

**Response:** We thank the reviewer for spotting this error; it has been corrected.

Hypotheses H2b (line 152) and H3c (line 160) are phrased in a way that is very hard to parse. Please consider rewriting.

**Response:** Thank you for this feedback. We agree that the phrasing of H2b and H3c could be clearer. To improve readability while adhering to our pre-registered analysis plan, we have rewritten the sentences to make the predicted effects easier to understand without changing their substance.

The revised text aims to more clearly articulate the predicted partner effects (H2b) and the interaction effects (H3c) on participant behaviour. The revised text now reads:

“Second, we predict that interactional indices will mediate the relationship between diagnostic status and rapport (H2). Specifically, (H2a) we predict that autistic individuals will display reduced behavioural indices (e.g. smiling) and high levels of kinematics indices (i.e., less smooth movements), leading to lower perceived rapport. We also predict a partner effect (H2b), where an actor’s rapport is influenced by their partner's behaviour; we hypothesise that an autistic partner will exhibit decreased social behaviours and altered kinematics, which in turn will reduce the actor’s perceived rapport. Finally (H2c), we predict that the shared diagnostic status of the dyad moderates these mediation effects. We suggest that the influence of social and kinematic cues on rapport will be strongest in non-autistic dyads and weakest in autistic dyads, with mixed-neurotype dyads falling in between.

Finally, we hypothesise that autistic participants will exhibit diminished behavioural indices and higher kinematic indices, than non-autistic participants (H3a), with these interactional indices further influenced by their partner’s diagnostic status (H3b). Finally (H3c), we predict an interaction between the diagnostic statuses of the dyad members will influence interactional indices. Specifically, we hypothesise that autistic participants will exhibit more interactional indices in mixed-neurotype dyads compared to when interacting with an autistic partner. This may be a response to the heightened social demands of masking or adapting their natural interaction style to align with perceived neurotypical norms. Full details and mediation models are provided in Supplemental Materials S2.”

In hypothesis H3a (line 158), I believe the authors mean to say that Autistic participants will exhibit dimished or reduced multimodal indices, as the amount of indices stays the same.

**Response:** Thank you for this important and precise suggestion. You are correct; our original wording was imprecise. We intended to predict a difference in the values of the indices, not the number of indices measured. We have revised the text to use the term "diminished" for greater clarity, which better reflects the intent of our pre-registered hypothesis.

Errant ")" on line 190.

**Response:** We thank the reviewer for spotting this error; it has been corrected.

Reviewer #2: Thank you for the opportunity to review this article. This is a well-written paper presenting a thoughtfully designed study that makes a valuable contribution to the field. The comprehensive analysis of verbal and non-verbal cues across same- and mixed-neurotype interactions addresses an important gap in the existing literature. I offer the following suggestions to enhance the paper's clarity.

**Response:** We thank the reviewer for their positive feedback on the manuscript. We appreciate their thoughtful suggestions to enhance the paper’s clarity.

Page 3, line 57: I find this sentence unclear. If a shift is mentioned, I would expect some contrast between views/methodologies to be present in the previous sentences, which is not the case.

**Response:** We thank the reviewer for highlighting this lack of clarity. We agree the contrast justifying the word 'shift' was not explicit enough. To address this, we have revised and merged the paragraphs one and two on page 3 (lines 42-58) to create a clearer argumentative flow that directly establishes this contrast. The text now reads:

“Social interactions are important for developing rapport, defined as a state of harmonious connection and mutual understanding between individuals[1,2]. This connection is developed through the intricate interplay of verbal and nonverbal communication and is crucial for forming social bonds and enhancing well-being[3–9]. Historically, research on social communication has focused on individual communicative elements like facial expressions, body language, and vocal tone in autistic and non-autistic populations[11–15]. However, these elements rarely occur in isolation during real-world interactions[16,17]. Instead, communication is inherently multimodal, with aspects like laughter seamlessly integrating with facial expressions, vocalisations, and body movements[18,19]. Furthermore, social interactions are dynamic, with adjustments to one person’s behaviour occurring in response to their partner[20,21]. Consequently, there has been a methodological shift towards studying dyadic (i.e. two-person) interactions to investigate the structural elements of conversation in non-autistic populations, including head nodding, facial expressivity, turn-taking, and gaze patterns, and their influence on conversational enjoyment and workplace success[22–24]. For example, smiles exchanged during conversations can both covertly and causally shape the inferences individuals draw about one another’s social intentions[25]. This shift towards understanding communication in naturalistic settings and social contexts is currently understudied in autistic adult populations.”

Page 6, lines 132-133: This sentence is slightly misleading. When first reading it, I assumed that utterance length had been measured in number of words, since it is introduced as a verbal interaction metric. However, the supplementary material reveals that length was measured in seconds, including silent pauses—which constitutes a non-verbal component. To avoid confusion, I suggest reformulating this sentence and/or specifying upfront that utterance length refers to the temporal duration of utterances.

**Response:** Thank you for this suggestion. We now introduce the concept of mean utterance length on page 3, line 55. On page 7 line 166-167 (which was formerly lines 132-133) we have amended this to state “mean utterance length (in seconds)” to avoid confusion. Please also see our response to your point below.

I would also like to see a brief explanation of 1) why utterance length was chosen as an interaction metric (utterance length is not mentioned in the Introduction, whereas other measures such as smiles, nods, movements are) and 2) why the authors chose to measure it in seconds rather than in words ?

**Response:** Thank you for this suggestion. We now introduce the concept of mean utterance length on page 3, line 55, and we have also included it on page 7 of the Introduction.

We chose to measure MUL in seconds, rather than words, as we were interested in MUL as a measure of conversational share and dynamics. MUL can be measured in words or morphemes which gives a good measure of linguistic complexity, but is less useful when considering conversational share and dynamics [26].

On page 23, line 608, in the limitations section, we now state: “Fifth, speech was quantified using mean utterance length in seconds. While this is a useful measure of conversational share and dynamics, we did not examine the linguistic content of what participants discussed, which may have influenced their rapport development.”

Page 6, line 136: I also wonder if referring to the eight elements of social interaction as multimodal indices is correct since most elements are not multimodal but unimodal (e.g., nods, smiling, total upper body velocity, acceleration, and jerkiness).

**Response:** Initially, we uses social behaviours and kinematic indices (see OSF pre-registration), we switched to using multimodal indices are a collective term for all outcome variables. But we understand the confusion that can arise with the term multimodal, we have replaced multimodal indices to interactional indices to avoid confusion yet still capture all outcomes in a single term. Further, when discussing the kinematics and manually coded behaviours we use the terms kinematic indices and behavioural indices, respectively. These changes have been made throughout the manuscript.

Page 7, lines 144-148: I find the formulation of hypothesis 1(and its sub-hypotheses) unclear and would suggest reformulating as separate sentences or as bullet points. Furthermore, it is not clear from the introduction and literature review why the authors hypothesise that self-reported rapport will be lower for autistic participants compared to non-autistic participants.

**Response:** We thank the reviewer for pointing this out. We have reformulated Hypothesis 1 and its sub-hypotheses into a clearer form. The rationale for hypothesising lower self-reported rapport in autistic participants was data-driven, based on the literature summarised in the introduction, which indicates that mixed-neurotype social interactions can present challenges that may influence perceptions of rapport.

Page 7, lines 149-164: Hypothesis 2a mentions social behaviors, are those the same as multimodal indices mentioned in Hypothesis 3? If yes, why use two different terms? If not, what does social behaviors refer to? Likewise, Hypothesis 2a stipulates that autistic individuals will produce increased, less smooth movements but Hypothesis 3a stipulates that they will exhibit fewer multimodal indices (which includes kinematic measures as stated on page 6 (lines 134-135) in the research aims). This seems contradictory.

Finally, the authors expect autistic participants to increase social behaviours such as smiling in mixed neurotype dyads due to heightened social demands (H3c). What does heightened social demands refer to specifically?

**Response:** We sincerely thank the reviewer for highlighting these critical issues. They are entirely correct that the manuscript was inconsistent and that the wording of our hypotheses created a confusing, apparent contradiction. We appreciate the opportunity to clarify our original intent and improve the manuscript.

The reviewer rightly questioned the contradiction. This was a failure of clarity in our writing, not in our underlying model. As stated in our pre-registration (OSF, H2a), our original hypothesis was specific and non-contradictory: we predicted that autistic individuals would exhibit lower levels of social behaviours (e.g., smiling) but higher levels of kinematic indices (e.g., faster, jerkier movements) as mediators of perceived rapport. We acknowledge that this crucial distinction was not clearly maintained in the submitted manuscript's text.

To resolve both the inconsistent terminology and the contradiction, we have thoroughly revised the manuscript around the clear, hierarchical framework we have now established. We now use the collective term interactional indices when referring to all outcome variables together. When discussing the specific categories, we consistently distinguish between:

Social indices: Our term for the manually coded behaviours (e.g., smiling, backchannelling).

Kinematic indices: Our term for the automated measures of motion (e.g., velocity, jerkiness).

This new framework is now used throughout the paper. It allows us to state our hypotheses unambiguously, matching our pre-registered plan: we predict lower social indices and higher kinematic indices for autistic individuals in specific contexts. This resolves the issues the reviewer rightly identified, and we are grateful for their careful reading.

I would suggest reformulating more clearly and consistently hypothesis 2 and 3 (and their sub-hypotheses).

**Response:** We have revise the hypotheses section, to be clearer.

Page 8, lines 185-186: The inclusion of self-diagnosed individuals in the autistic group should be mentioned upfront in the abstract. While I fully support including self-identified participants—given recruitment challenges and barriers to formal diagnosis—this information needs to be transparent from the outset. Group composition impacts result interpretation and generalizability and should also be discussed in the Discussion/Limitation section.

**Response:** Thank you for this important point on transparency. We have now added a note to the abstract specifying the inclusion of self-diagnosed participants. We have also expanded the Limitations section of our Discussion to address the potential impact of this on the interpretation and generalisability of our findings.

Related to this, I also wonder if it is appropriate to use “diagnostic status” as outcome variable name, given the mixed status of participants. Maybe a preliminary note would be useful to clarify what the authors mean by diagnostic status.

**Response:** Thank you for this thoughtful point regarding terminology. We agree that the term 'diagnostic status' could be interpreted as referring exclusively to clinical diagnoses, and we appreciate the opportunity to clarify our usage. To address this, we have added a sentence in the Methods section where the term is first introduced. This note explains that, for the purposes of this paper, 'diagnostic status' is used as an umbrella term to refer to participants' identification as either autistic (whether through clinical or self-diagnosis) or non-autistic. This ensures our terminology is clear and consistent with our inclusive approach to participant recruitment. On page 9, lines 225-227 it now reads: ‘In this paper, 'diagnostic status' is used as a collective term to refer to an individual's identification as autistic (either through formal clinical diagnosis or self-diagnosis) or non-autistic.’

I'd also recommend reporting the distribution of clinical versus self-diagnosis across dyad types, not just by individual group membership.

**Response:** Thank you for this valuable suggestion. We have now added information to the manuscript regarding the distribution of clinical versus self-diagnosed autistic participants across the relevant dyad types. A Pearson's Chi-squared test confirmed that the proportion of clinically-diagnosed to self-diagnosed participants did not differ significantly between the autistic dyads and the mixed dyads (χ²(1, N = 54) = 0.26, p = .61). This information has been added to the Methods section to confirm that diagnosis method was not a confounding factor. We have added the group count to Table 1.

Page 8, line 229: Could the authors provide more information on how dyad partners were matched. Did they try to match them by age or sex/gender?

**Response:** Thank you for this question. We have clarified this point in the manuscript. The matching of dyad partners was semi-random. Where possible, we aimed to minimise gender mismatch between partners. However, as pairings were ultimately determined by the available pool of participants on each day of testing, this was not a strict rule. We clarified this on page 9, line 268-270, which now reads:

“Dyad partners were matched semi-randomly based on participant availability on each day of testing, with an aim to minimise gender mismatch where possible.”

Page 11, line 254: What were the causes of coding disagreement for the variable “Smiling” ? Were they systematic or random ? This result should also be mentioned in the Discussion or Limitation section.

**Response:** We thank the reviewer for this important question. The disagreements were not systematic but arose from specific ambiguities inherent in coding naturalistic interactions. These included challenges in distinguishing the start and end points of smiles that occurred between laughs, and suboptimal camera angles where participants were not directly facing the camera, resulting in only partial visibility of the mouth. We have now added a sentence to the Limitations section to acknowledge that while our overall interrater reliability for this variable was good, this ambiguity remains a potential source of noise in the data. Page 23, lines 638-645 now includes:

“It should also be noted that the behavioural coding for smiling, while reliable, was subject to some ambiguity; suboptimal camera angles at times resulted in only partial facial visibility, and coders occasionally faced challenges in differentiating subtle smiles from laughter, which may have introduced noise into this specific measure.”

Typos
Page 5, 99-99: To address this gap, the Actor-Partner Interdependence Model (APIM) is a is a statistical model used to analyse data from dyadic relationships which allows for the simultaneous examination of how each person's behaviours and characteristics influence not only their outcomes but those of their partner.

**Response:** We thank the reviewer for spotting this error; it has been corrected.

Page 12, line 276: recording --> recoding

**Response:** We thank the reviewer for spotting this error; it has been corrected.

**References**

1.	Hagad JL, Legaspi R, Numao M, Suarez M. Predicting Levels of Rapport in Dyadic Interactions through Automatic Detection of Posture and Posture Congruence. 2011 IEEE Third International Conference on Privacy, Security, Risk and Trust and 2011 IEEE Third International Conference on Social Computing. 2011. pp. 613–616. doi:10.1109/PASSAT/SocialCom.2011.143

2.	Puccinelli NM, Tickle-Degnen L, Rosenthal R. Effect of Dyadic Context on Judgments of Rapport: Dyad Task and Partner Presence. JOURNAL OF NONVERBAL BEHAVIOR.

3.	Alsarrani A, Hunter RF, Dunne L, Garcia L. Association between friendship quality and subjective wellbeing among adolescents: a systematic review. BMC Public Health. 2022;22: 2420. doi:10.1186/s12889-022-14776-4

4.	Hall JA, Horgan TG, Murphy NA. Nonverbal Communication. Annual Review of Psychology. 2019;70: 271–294. doi:10.1146/annurev-psych-010418-103145

5.	Orben A, Tomova L, Blakemore S-J. The effects of social deprivation on adolescent development and mental health. The Lancet Child & Adolescent Health. 2020;4: 634–640. doi:10.1016/S2352-4642(20)30186-3

6.	Bernieri FJ, Davis JM, Rosenthal R, Knee CR. Interactional Synchrony and Rapport: Measuring Synchrony in Displays Devoid of Sound and Facial Affect. Pers Soc Psychol Bull. 1994;20: 303–311. doi:10.1177/0146167294203008

7.	Cross L, Atherton G, Sebanz N. Intentional synchronisation affects automatic imitation and source memory. Sci Rep. 2021;11: 573. doi:10.1038/s41598-020-79796-9

8.	Tunçgenç B, Cohen E, Fawcett C. Rock With Me: The Role of Movement Synchrony in Infants’ Social and Nonsocial Choices. Child Development. 2015;86: 976–984. doi:10.1111/cdev.12354

9.	Lakens D, Stel M. If They Move in Sync, They Must Feel in Sync: Movement Synchrony Leads to Attributions of Rapport and Entitativity. Social Cognition. 2011;29: 1–14. doi:10.1521/soco.2011.29.1.1

10.	McNaughton KA, Moss A, Yarger HA, Redcay E. Smiling synchronization predicts interaction enjoyment in peer dyads of autistic and neurotypical youth. Autism. 2024; 13623613241238269. doi:10.1177/13623613241238269

11.	Mello M, Fusaro M, Aglioti SM, Minio-Paluello I. Exploring social touch in autistic and non-autistic adults via a self-report body-painting task: The role of sex, social context and body area. Autism. 2024; 13623613231218314. doi:10.1177/13623613231218314

12.	Philip RCM, Whalley HC, Stanfield AC, Sprengelmeyer R, Santos IM, Young AW, et al. Deficits in facial, body movement and vocal emotional processing in autism spectrum disorders. Psychological Medicine. 2010;40: 1919–1929. doi:10.1017/S0033291709992364

13.	Atkinson AP, Dittrich WH, Gemmell AJ, Young AW. Emotion Perception from Dynamic and Static Body Expressions in Point-Light and Full-Light Displays. Perception. 2004;33: 717–746. doi:10.1068/p5096

14.	Bänziger T, Patel S, Scherer KR. The Role of Perceived Voice and Speech Characteristics in Vocal Emotion Communication. J Nonverbal Behav. 2014;38: 31–52. doi:10.1007/s10919-013-0165-x

15.	Efthimiou TN, Baker J, Clarke A, Elsenaar A, Mehu M, Korb S. Zygomaticus activation through facial neuromuscular electrical stimulation (fNMES) induces happiness perception in ambiguous facial expressions and affects neural correlates of face processing. Social Cognitive and Affective Neuroscience. 2024;19: nsae013. doi:10.1093/scan/nsae013

16.	Patterson ML. A Systems Model of Dyadic Nonverbal Interaction. J Nonverbal Behav. 2019;43: 111–132. doi:10.1007/s10919-018-00292-w

17.	Hamilton AF de C, Holler J. Face2face: advancing the science of social interaction. Philosophical Transactions of the Royal Society B: Biological Sciences. 2023;378: 20210470. doi:10.1098/rstb.2021.0470

18.	Bohy H, Hammoudeh A, Maiorca A, Dupont S, Dutoit T. Analysis of Co-Laughter Gesture Relationship on RGB videos in Dyadic Conversation Contex. arXiv; 2022. Available: <http://arxiv.org/abs/2205.10266>

19.	Jokinen K, Trong TN, Wilcock G. Body movements and laughter recognition: experiments in first encounter dialogues. Proceedings of the Workshop on Multimodal Analyses enabling Artificial Agents in Human-Machine Interaction. New York, NY, USA: Association for Computing Machinery; 2016. pp. 20–24. doi:10.1145/3011263.3011264

20.	Kenny DA. Reflections on the actor–partner interdependence model. Personal Relationships. 2018;25: 160–170. doi:10.1111/pere.12240

21.	Kenny DA, Malloy TE. Partner effects in social interaction. J Nonverbal Behav. 1988;12: 34–57. doi:10.1007/BF00987351

22.	Deeb H, Leal S, Vrij A, Mann S, Dabrowna O. To Nod or Not to Nod: How Does Interviewer Nonverbal Behavior Affect Rapport Perceptions and Recall in Truth Tellers and Lie Tellers? J Nonverbal Behav. 2024;48: 25–45. doi:10.1007/s10919-024-00452-1

23.	Kavanagh E, Whitehouse J, Waller BM. Being facially expressive is socially advantageous. Sci Rep. 2024;14: 12798. doi:10.1038/s41598-024-62902-6

24.	Stivers T. Is Conversation Built for Two? The Partitioning of Social Interaction. Research on Language and Social Interaction. 2021;54: 1–19. doi:10.1080/08351813.2020.1864158

25.	Arias-Sarah P, Bedoya D, Daube C, Aucouturier J-J, Hall L, Johansson P. Aligning the smiles of dating dyads causally increases attraction. Proc Natl Acad Sci USA. 2024;121: e2400369121. doi:10.1073/pnas.2400369121

26.	Richardson D, Dale R, Shockley K. Synchrony and swing in conversation: Coordination, temporal dynamics, and communication. Embodied communication in humans and machines. 2008; 75–94.
